# *Bacillus subtilis* spores displaying *Toxoplasma gondii* GRA12 induce immunity against acute toxoplasmosis

**DOI:** 10.3389/fimmu.2025.1457560

**Published:** 2025-02-26

**Authors:** Hong-chao Sun, Xiu-fang Yuan, Wei Zhou, Zhi-jin Zhou, Fei Su, Yuan Fu, Li-li Hao, Xin Liu, Xin Zhou, Shi-yi Ye, Li-hua Xu, Bin Yu, Jun-xing Li, Tuan-yuan Shi

**Affiliations:** ^1^ Institute of Animal Husbandry and Veterinary Science, Zhejiang Academy of Agricultural Sciences, Hangzhou, Zhejiang, China; ^2^ Zhejiang Center of Animal Disease Control, Hangzhou, Zhejiang, China; ^3^ Department of Animal Parasitology, College of Animal and Veterinary Sciences, Southwest Minzu University, Chengdu, Sichuan, China

**Keywords:** toxoplasmosis, GRA12, *Bacillus subtilis*, vaccine, protective efficacy

## Abstract

**Background:**

*Toxoplasma gondii* (*T. gondii*) is a widely prevalent intracellular parasite that infects almost all warm-blooded animals and causes serious public health problems. The drugs currently used to treat toxoplasmosis have the disadvantage of being toxic and prone to the development of resistance, and the only licensed vaccine entails a risk of virulence restoration. The development of a safe and effective vaccine against *T. gondii* is urgently needed. *Bacillus subtilis* (*B. subtilis*) has been used as a potential vaccine expression vector for the treatment and prevention of various diseases. *T. gondii* GRA12 is a key virulence factor that resists host innate immunity and exhibits good antigenicity with several excellent B and T cell epitopes.

**Methods:**

A recombinant spore named rBS-GRA12 was constructed by fusing the *T. gondii* GRA12 protein to the *B. subtilis* coat protein B (CotB). rBS-GRA12 spores were identified by PCR, western blotting, immunofluorescence assays, amylase activity, and ultrastructural analysis. Immunological experiments were then conducted to assess the immunoprotective effects of rBS-GRA12. Groups of mice immunized with rBS-GRA12 (10^6^, 10^8^, or 10^10^ colony-forming units), GRA12 protein emulsified with Freund’s adjuvant (FA+GRA12), Freund’s adjuvant alone (FA), phosphate buffered saline (PBS), or wild-type *B. subtilis* spores (WT). Splenocyte proliferation, antibodies, and cytokine expression levels were used to assess immune responses induced by the immunizations. All groups were inoculated with *T. gondii* RH strain, and survival times and parasite loads in tissues were used to assess protective effects against *T. gondii* infection.

**Results:**

Amylase activity assays confirmed the generation of recombinant *B. subtilis*. PCR, western blotting and immunofluorescence assays confirmed that the rBS-GRA12 spores expressed GRA12. Observation of rBS-GRA12 spores via transmission and scanning electron microscopy indicated that GRA12 expression had no effect on spore morphology or structure. Splenocyte proliferation was significantly greater in all three rBS-GRA12 groups than in the FA+GRA12 group, and IgG and IgG2a subclass titers were higher. Substantial production of interferon gamma (IFN-γ), interleukin (IL)-12, and an increase in IL-4 production were evident in the rBS-GRA12-10^8^ group. Secretory sIgA levels were significantly elevated in all three rBS-GRA12 groups than in the FA+GRA12 group and the control groups. Brain and liver tissues parasite loads were significantly lower in the three rBS-GRA12 groups than in any other group. Compared to all other groups, mice in the three rBS-GRA12 groups exhibited longer survival times when challenged with acute *T. gondii* infection.

**Conclusion:**

Mice immunized with rBS-GRA12 exhibited higher levels of cellular, humoral, and mucosal immune responses than control mice. These results provide a new perspective for the development of *T. gondii* vaccines.

## 1 Introduction

*Toxoplasma gondii* (*T. gondii*) is an obligate intracellular protozoan parasite that infects nearly one third of the world’s population (1). Its life cycle is complex, involving a sexual stage in feline hosts and an asexual stage in intermediate hosts (2). *T. gondii* exists mainly as tachyzoites (acute phase and clinical manifestations), and bradyzoites (chronic phase) in the intermediate host. *T. gondii* infection is acquired by consumption of oocysts shed by cats in contaminated water or food, or by ingestion of meat containing tissue cysts, as well as by congenital infection (3, 4). Although usually asymptomatic in immunocompetent adults, immunocompromised patients exhibit significant clinical symptoms after infection, and even death (5). Infection during pregnancy can lead to fetal infection, resulting in miscarriage or stillbirth (6). *T. gondii* is an opportunistic pathogen. Porcine reproductive and respiratory syndrome virus and classical swine fever virus can increase the likelihood of pigs becoming infected with *T. gondii*, and both are commonly found co-existing with toxoplasmosis in pigs in China (7). In a recently reported case, a 60-year-old man presented with severe ocular lesions due to *Epstein Barr Virus* and *T. gondii* coinfection (8). A rare pattern of iridian atrophy with multiple areas of “polka dots” has also been reported in association with *varicella-zoster* virus and *T. gondii* coinfection (9). In livestock, particularly sheep and goats, toxoplasmosis can cause mortality resulting in huge losses (10).

Conventional drugs against *T. gondii* have historically been associated with toxic effects and the development of drug resistance (11). They are only effective against tachyzoites, and have no effects on encysted bradyzoites (12). Vaccination is theoretically the best way to control *T. gondii*, but no human vaccine is available. The commercially available live attenuated vaccine “Toxovax”, derived from the S48 *T. gondii* tachyzoite, is currently only licensed for use in sheep to prevent abortion (13). Notably however, Toxovax does not provide complete protection against toxoplasmosis, with 72.3% and 80.8% of lambs/fetuses surviving after vaccination with S48 tachyzoites passed through the peritoneal cavity of mice or Vero cell culture (14). It is also expensive, and can be transformed into pathogenic forms (15). The search for a safe and effective *T. gondii* vaccine has been a major focus of research. Several studies have investigated various immunization strategies, including the use of subunits (protein orDNA) and live attenuated vaccine agents, but subunit vaccines have only provided partial immunological protection, and there is a risk of virulence enhancement associated with live attenuated vaccines (16). New approaches are needed to develop effective *T. gondii* vaccines.

*T. gondii* secretes large amounts of dense granule (GRA) proteins upon host invasion, which are involved in the formation of parasitophorous vacuoles and network structures that enable the parasite to replicate (17). GRA proteins are some of the most promising vaccine candidates against *T. gondii* infection (18), particularly GRA12 which is present in the GRA core complex within dense granules (17). The GRA12 protein evidently contains 436 amino acid residues, 53 post-translational modification sites, and several potential B and T cell epitopes. Antigenicity and allergenicity investigations indicate that GRA12 is immunogenic and non-allergenic (19), and these data suggest that GRA12 is an excellent vaccine candidate antigen. Live attenuated vaccines tend to be very effective because they induce both cellular and humoral immune responses, and the strains used are usually chosen after screening for naturally occurring and/or artificially generated attenuation. The development of vaccine strains is fraught with uncertainty and long development cycles, and a risk of regression of attenuated strains (20). A live vector vaccine has the advantages of the good safety of an inactivated vaccine, the good immunogenicity and low cost of a live vaccine, and a reduced risk of virulence regression. Humoral, cellular and mucosal immunity can be induced by live vector vaccines (21).

*Bacillus subtilis* (*B. subtilis*) is considered a good candidate for exogenous gene expression. It is safe, non-toxic, and can survive under harsh environmental conditions (22). It is also able to produce endospores when nutrition is scarce, and mature *B. subtilis* spores can survive in a dormant form for long periods, enabling the species to endure protracted adverse events. Since the method for producing competent *B. subtilis* was established in 1958, the *B. subtilis* expression system has attracted much attention (23). *B. subtilis* WB800n is a genetically modified *B. subtilis 168* variant that has all extracellular proteases knocked out, enabling it to be used for the expression of secretory proteins in a wide range of applications. Cysteine protease from *Clonorchis sinensis* has been successfully expressed on the surface of *B. subtilis* spores, and the degree of liver fibrosis was significantly reduced after oral immunization with these recombinant spores (24). *B. subtilis* spores are surrounded by a coat, and coat protein B (CotB) is one of the outer coat proteins considered a suitable fusion partner for the display of heterologous antigens (25). In the current study CotB was used as a fusion protein to generate a recombinant *B. subtilis* strain expressing *T. gondii* GRA12 on its spore surface. The capacity of this recombinant spore-based vaccine to induce an immune response and provide protection against *T. gondii* was assessed via oral immunization.

## 2 Materials and methods

### 2.1 Mice and ethics approval statement

BALB/c mice aged 6–8 weeks of age were obtained from the Laboratory Animal Center of the Zhejiang Academy of Agricultural Sciences. All procedures including the use of Freund’s complete adjuvant) were approved by the Animal Ethics Committee of Zhejiang Academy of Agricultural Sciences in accordance with the recommendations of the National Institutes of Health Guide for the Care and Use of Laboratory Animals (Ethics Protocol No. 2023ZAASLA67). Mice were housed under specific pathogen-free conditions at 50%–60% humidity and 22°C,and had free access to food and water.

### 2.2 Cell culture and parasite, *B. subtilis* and *T. gondii* lysate antigens

Vero cells and *T. gondii* RH strain tachyzoites were obtained from Professor Aifang Du’ laboratory at Zhejiang University and were stored in liquid nitrogen at our laboratory. *B. subtilis 168 and B. subtilis* WB800n strains were purchased from Wuhan Miaoling Biotechnology.

Vero cells were maintained in Dulbecco’s modified Eagle’s medium (DMEM) supplemented with streptomycin (100 μg/mL), penicillin (100 U/mL) and 10% fetal bovine serum (FBS). Cells were cultured at 37°C in a humidified 5% CO_2_ atmosphere. *T. gondii* RH strain Tachyzoites were cultured in Vero cells which were cultured in DMEM supplemented with 10% inactivated FBS. The tachyzoites were harvested by passing the culture preparation through a 27-gauge needle 3–5 times. Tachyzoites were then centrifuged and washed three times with sterile phosphate buffered saline (PBS) to remove cellular debris. These purified tachyzoites were disrupted by freezing at -80°C and thawing at room temperature three times, followed by sonication at 400 W for 5 min. The supernatant of the parasite lysate was harvested via centrifugation at 10, 000 g for 30 min at 4°C, and *T. gondii* lysate antigen (TLA) concentration was measured via a BCA protein assay kit (26).

### 2.3 Gene fusion vector construction

The promoter sequence (263 bp) and a partial N-terminal coding DNA sequence (825 bp) of the *CotB* gene were amplified from genomic *B. subtilis* strain 168 DNA via PCR. The primers used were 5’-CG *GGATCC* acggattaggccgtttgtcc-3’ which contains a Bam*HI* restriction enzyme site (forward), and 5’- CC *AAGCTT* GGATGATTGATCATCTGAAG-3’ which contains a Hind*III* restriction enzyme site (reverse). The GRA12 coding DNA sequence was amplified from the total cDNA of *T. gondii* RH tachyzoites using the primers 5’-CC *AAGCTT* atgCGACATGTTGGCGGTTTCTCGG-3’ (forward) and 5’-GC *GAATTC* TCAGTTGTGTTTGCTGCCTGCAGAG-3’ (reverse), in which EcoR*I* and Hind*III* restriction sites were inserted (represented by italics). The PCR products of *CotB* and GRA12 were inserted into the pDG364 vector (Miaoling Bio, China), and the recombinant *CotB-GRA12-pDG364* plasmid was obtained. The recombinant plasmid was verified via DNA sequencing to ensure that all gene sequences were correct (no mutations or losses). (Sangon Biotech, Shanghai, China).

### 2.4 Electroporation, preparation, and identification of the recombinant spore

The recombinant plasmid was digested with the restriction enzyme Xba*I* and transformed into *B. subtilis* WB800n strain as previously described (23, 27). The products were cultured in LB plates with 5 μg/mL chloramphenicol for 24–36 h. Clones were identified via amylase activity analysis, PCR and DNA sequencing. Approximately 3–5 μL of recombinant *B. subtilis* was cultured in LB with 1% soluble starch and without starch, and wild-type *B. subtilis* was used as a negative control. After incubation overnight, iodine solution was added to each plate and the color reactions were assessed.

Sporulated spores and outer layer proteins were prepared as previously described (28, 29). Briefly, recombinant *B. subtilis* was recovered and cultured in DSM medium (1:1000) for 48 h, then centrifuged to facilitate precipitate collection. The samples were then treated with 1 mM phenylmethylsulfonyl fluoride and 0.02 g/L lysozyme for 30 min. The spores were then washed with 1 M NaCl and 1M KCl and washed twice with distilled water. Finally the spores thus obtained were resuspended in sterile water and incubated at 68°C for 1 h.

Spores were treated with spore chlamydosin extraction buffer for 2 h, washed six times with 1 M Tris-HCl buffer (pH 8.0), then suspended in 5 mL sonication buffer and sonicated for 10 min. Coat proteins were collected from precipitates after centrifugation. Extracted proteins were loaded onto 12% sodium dodecyl sulfate polyacrylamide gels. Polyclonal antibody harvested from GRA12- immunized mice diluted 1:1000 was used as the primary antibody (30), and HRP-conjugated sheep anti-mouse IgG (Solarbio, Beijing, China) diluted 1:2000 was used as the secondary antibody. The GRA12 recombinant protein was injected intraperitoneally into mice aged 6-8 weeks to generate polyclonal antibody (100 μg/mouse for the first immunization and 50 μg/mouse for the second and third immunizations). Finally, polyvinylidene fluoride filter membranes were observed via enhanced chemiluminescence reagent (Sangon Biotech, Shanghai, China).

Immunofluorescence assays were performed to validate GRA12 protein expression as previously described, with a minor modification (31). Briefly, *B. subtilis* spores were blocked with 5% bovine serum albumin (BSA) in PBS for 1 h at 37°C,then incubated with mouse anti-GRA12 polyclonal antibody (1:1000 dilution, prepared and stored in our laboratory). After washing three times with PBS, *B. subtilis* spores were stained with Alexa Fluor 488-conjugated goat anti-mouse IgG (Beyotime, China, 1: 500 dilution), then observed and photographed using an Olympus fluorescence microscope (DP71, Japan).

### 2.5 Scanning electron microscopy and transmission electron microscopy analysis

*B. subtilis* spores were prepared as described previously with a minor modification (32, 33). Briefly, wild-type and recombinant *B. subtilis* strains were inoculated into LB medium and cultured overnight, followed by inoculation into DSM medium at a ratio of 1:100 and incubation at 37°C for 72 h. The *B. subtilis* was then centrifuged at 10, 000 g and the supernatant were discarded, then the precipitate was washed three times with sterile deionized water and resuspended in deionized water. Lysozyme was added to a final concentration of 0.02 g/L in a water bath at 37°C for 2 h, followed by sequential washes with 1 mol/L sterile NaCl and 1 mol/L sterile KCl. Spores were obtained via resuspension in sterile deionized water.

For scanning electron microscopy (SEM), spores were collected and fixed in 2.5% glutaraldehyde solution at 4°C overnight. After centrifugation, the fixative was discarded, the spores were washed three times with 0.1 mol/L sterile PBS, and fixed with 1% osmium tetroxide for 2 h. Dehydration was performed sequentially with 30%, 50%, 70%, 80%, 90%, 95%, and 100% ethanol solutions for approximately 15 to 20 min at each step after three washes with sterile PBS. The samples were then transferred into a 1:1 mixture of alcohol and iso-amyl acetate for approximately 30 min, then to pure iso-amyl acetate for approximately 1 h. Lastly, the samples were dehydrated in a Hitachi Model HCP-2 critical point dryer with liquid CO_2_, then coated with gold-palladium and examined via SEM (Hitachi Regulus 8100).

For transmission electron microscopy (TEM), spores were treated using the same steps described above, then transferred to absolute acetone for 20 min after dehydration via a graded series of ethanol (50%, 70%, 80%, 90%, 95% and 100%) for approximately 15 to 20 min. Specimens were then placed in a 1:1 mixture of absolute acetone and embedding medium for 1 h, then transferred to a 1:3 mixture of acetone and embedding medium for 3 h, and lastly to the final embedding medium overnight. The specimens were then placed in capsules containing embedding medium and heated at 70°C for 9 h. Specimen sections were stained with uranyl acetate and alkaline lead citrate for 15 min, and examined via TEM (Hitachi H7650).

### 2.6 Immunization and challenge

Seven groups of mice (20 mice/each group) were immunized. The seven groups were immunized with rBS-GRA12 (10^6^, 10^8^, or 10^10^ colony-forming units [CFU]), GRA12 protein emulsified with Freund’s adjuvant (FA+GRA12; 100 μL), Freund’s adjuvant alone (FA; 100 μL), PBS (100 μL), or wild-type *B. subtilis* spores (WT; 10^10^ CFU). Spore numbers were calculated via a previously described gradient dilution method (34), with minor modifications. Briefly, *B. subtilis* were grown in DSM at 37°C for 48 to 72 h and the culture was serially diluted in PBS (1:10) supplemented with MgSO_4_ (1 mM) six times, then 100 µL aliquots of the dilution were incubated on a DSM agar plate. The dilution was incubated at 80°C for 20 min, and 100 µL aliquots of the heat-treated dilution were inoculated onto DSM agar plates. CFU counts were determined after overnight incubation at 37°C.

Mice in the PBS, WT, and recombinant spores’ groups were gavaged for 3 consecutive days, followed by two boosters at 1-week intervals. Each boost was administered the same way as the original immunization. GRA12 recombinant protein was emulsified with Freund’s complete adjuvant at a 1:1 ratio, then mice were intraperitoneally immunized with GRA12 protein (200 μg/mouse), followed by two boosters of 100 μg of GRA12 emulsified with Freund’s incomplete adjuvant administered intraperitoneally 2 weeks apart. Mice in the FA group were initially immunized intraperitoneally with complete Freund’s adjuvant, then incomplete Freund’s adjuvant was used for the second and third immunizations. A description of animal grouping is shown in **Supplementary Table S1** and **Supplementary Figure S1** (the **Supplementary Material**). Five weeks after the last injection, mice were challenged intraperitoneally with 10^4^ *T. gondii* RH strain tachyzoites, and survival time was monitored.

In order to evaluate parasite loads, brain and liver tissues were collected from three mice in each group that exhibited clinical symptoms but did not die. The experiment was performed via absolute fluorescence quantitative PCR using primers specific for the *T. gondii* B1 gene (F, 5’- GGAACTGCATCCGTTCATGAG-3’; R, 5’-TCTTTAAAGCGTTCGTGGTC-3’) and an Applied Biosystems Inc. 7500 fluorescence quantitative PCR instrument. Numbers of parasites in tissue samples were calculated via a standard curve for the amplification of *T. gondii* genomic DNA templates at known concentrations (35). Experimental data were obtained from three replicate experiments.

### 2.7 Determination of antibodies via enzyme-linked immunosorbent assays

Blood samples were collected from the venous plexus of mouse tails, and serum samples were obtained. *T. gondii*-specific serum antibody levels were measured via enzyme-linked immunosorbent assays (ELISAs) in accordance with the manufacturer’s instructions (MultiSciences, Hangzhou, China). Briefly, 100 μL of TLA (20 μg/mL) was added to a 96-well plate and incubated overnight at 4°C, then the plate was blocked with 2% BSA in PBS for 2 h at 37°C. After three washes with PBS containing 0.05% tween-20 (PBST), mouse sera (1:100 diluted with 1% BSA in PBS) were added to the plate, followed by incubation at 37°C for 2 h. After three washes with PBST, horseradish peroxidase (HRP)-conjugated anti-mouse IgG, IgG1, and IgG2a (1:2000 diluted in 1% BSA in PBS) were added to designated wells for 40 min at 37°C. Tetramethylbenzidine substrate solution was added for 15 min in the dark, then the reaction was stopped with 2 M H_2_SO_4_ and the absorbance was read at OD 450 nm using an ELISA reader. Each sample was assayed in triplicate.

Small intestinal lavage fluids samples were collected from each group as previously described (36). These fluids were assessed via ELISA at a starting dilution of 1:2, and HRP-conjugated anti-mouse IgA (1:1000, Sanying Biotechnology, Wuhan, China) was used as the secondary antibody. The other steps used were the same as those described above for IgG detection.

### 2.8 Lymphocyte proliferation assay and cytokine assays

Spleens were removed from three mice in each group 1 day before *T. gondii* challenge, then ground with a sterile grinding rod and filtered through a nylon sieve. Splenic lymphocytes suspensions were collected and red blood cells (RBCs) were removed using RBC lysis buffer (Solarbio, Beijing, China). After three washes with DMEM medium, splenic lymphocytes (10^5^/well) were plated into 96-well plates and cultured with TLA (20 μg/mL), or 7.5 μg/mL concanavalin A (ConA, Sigma, USA) as a positive control, or DMEM medium alone (negative control) at 37°C in 5% CO_2_ for 72 h. Cell proliferation was measured using a CCK-8 cell counting kit in accordance with the manufacturer’s instructions (Biosharp, Anhui, China). Absorbance was measured at OD 450 nm.

For cytokine assay, splenocytes were harvested as described above and cultured with TLA (20 μg/mL) or DMEM medium alone at 37°C in 5% CO_2._ Cell culture supernatants were harvested, and interleukin (IL)-2 and IL-4 concentrations were assessed at 24 h, and interferon gamma (IFN- γ) and IL-12 were assessed at 72 h and 96 h, in accordance with established recommendations (MultiSciences, Hangzhou, China). Levels of IL-2, IL-4, IFN- γ, and IL-12 were also assessed in the sera of three mice from each group via ELISA method in accordance to the manufacturer’s instructions (MultiSciences, Hangzhou, China).

### 2.9 Statistical analysis

Statistical analyses were performed using SPSS Statistics version 26.0 and GraphPad Prism 5 (GraphPad Software). A normality test has been performed and the data have a normal distribution. One-way analysis of variance was used to analyze differences in antibody and cytokine levels, and Student’s t test was used for statistical comparisons between two groups. The Kaplan-Meier approach was used to assess survival. *P* < 0.05 was deemed to indicate statistical significance.

## 3 Results

### 3.1 Recombinant CotB-GRA12-pDG364 plasmid construction

**Figure 1:**
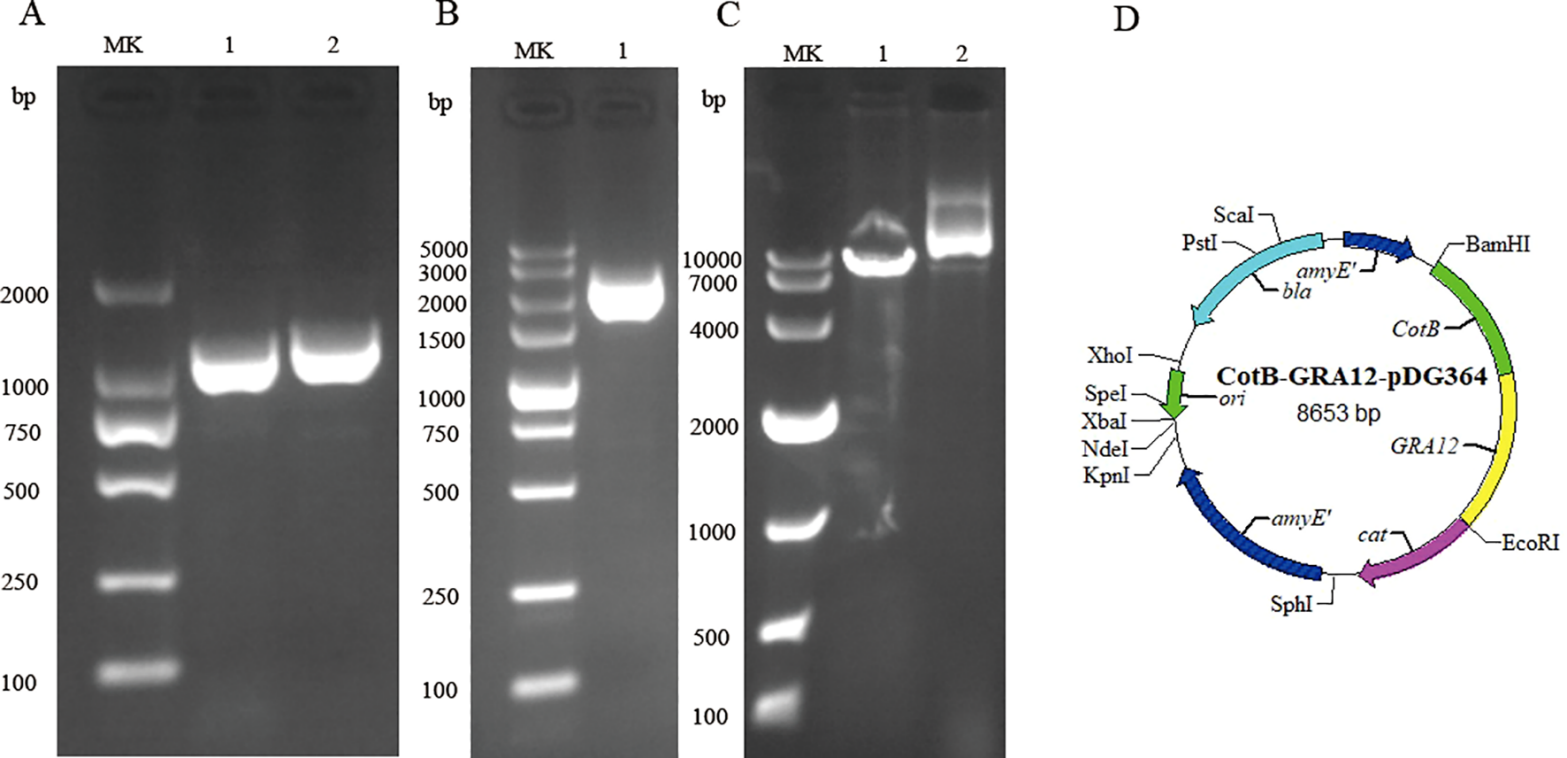
Construction of the recombinant CotB-GRA12-pDG364 plasmid. **(A)** PCR amplification of the *B. subtilis* CotB gene and the *T. gondii* GRA12 gene. MK, Takara DL 2000 DNA marker. Lane 1, *B. subtilis* CotB. Lane 2, *T. gondii* GRA12. **(B)** Overlap PCR amplification of the CotB-GRA12 fusion gene. MK, Takara DL 5000 DNA marker. Lane 1, the target CotB-GRA12 band. **(C)** Identification of the recombinant plasmid using a single enzyme digest. MK, Takara DL 10000 DNA marker. Lane 1, linearized fragment. Lane 2, control of plasmid. **(D)** Mapping of the CotB-GRA12-pDG364 plasmid.

The *B. subtilis* strain 168 genome was used as a template to amplify the CotB gene of the *B. subtilis* capsid protein. The cDNA of *T. gondii* (RH strain, type I representative strain) was used as a template to amplify the GRA12 gene. The PCR amplification products of the CotB and GRA12 genes were subjected to 1.5% agarose gel electrophoresis. The amplified fragments were consistent with the theoretically predicted sizes of the target bands (**Figure 1A**). Sequenced of PCR products exhibited 100% similarity with the target sequence. The CotB-GRA12 fusion gene was successfully amplified using the amplified products of CotB and GRA12 as a template (**Figure 1B**), and sequencing results indicated that fusion gene sequences were identical to those of the target gene. The CotB-GRA12 fusion gene was ligated to the pDG364 plasmid using the Seamless Cloning Kit, and after linearization of the recombinant plasmid, the target bands obtained were of the expected size (**Figure 1C**). CotB-GRA12-pDG364 mapping is shown in **Figure 1D**.

### 3.2 Identification of recombinant *B. subtilis*

**Figure 2:**
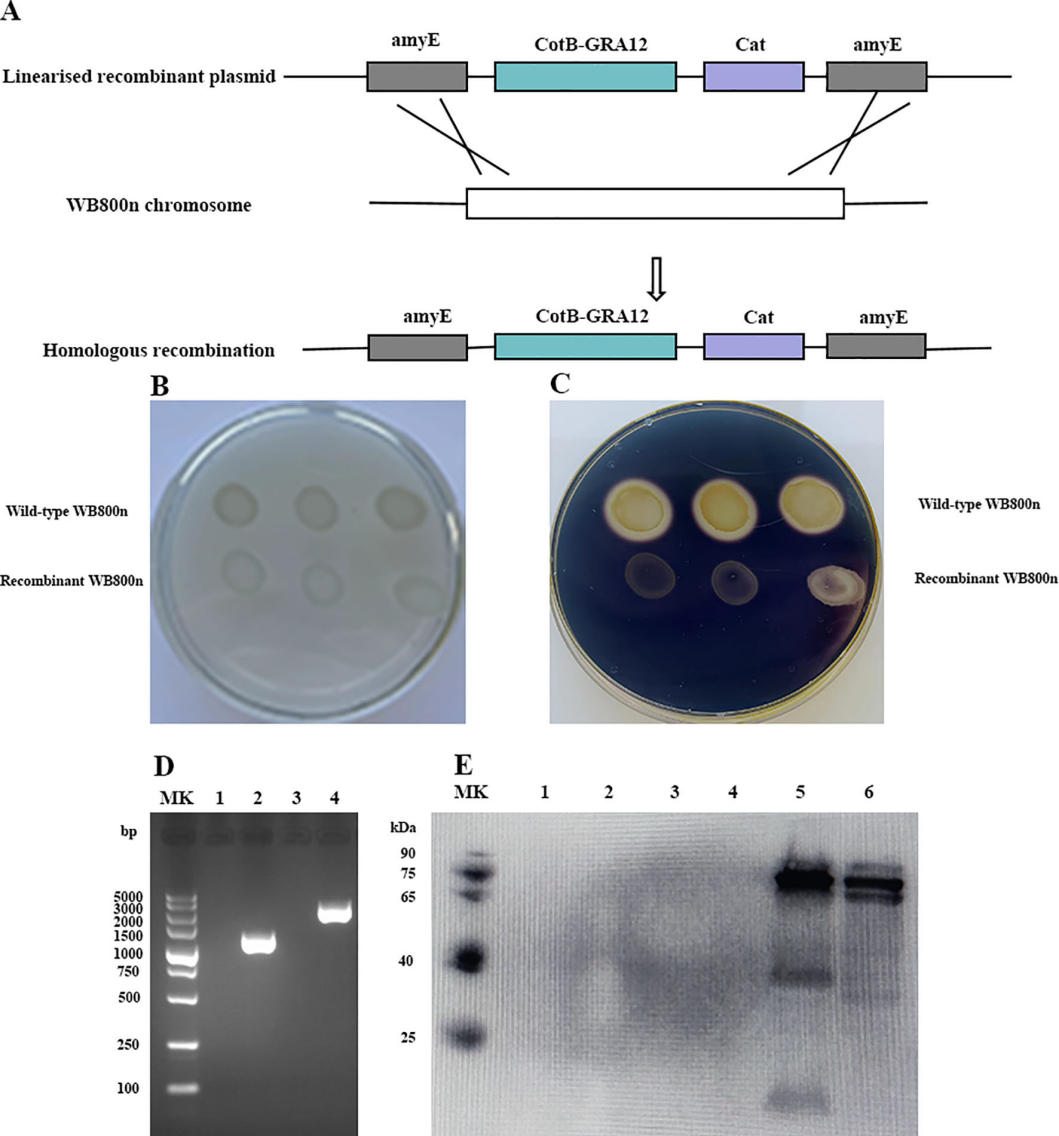
Preparation and identification of the recombinant *B. subtilis* spore. **(A)** Flowchart representing the construction of recombinant *B. subtilis*. **(B, C).** Identification of amylase activity. **(B)** The control group without iodine solution. **(C)** Addition of iodine solution to elicit a color reaction. Each plate contained a wild-type and a recombinant strain of *B. subtilis*. **(D)** PCR amplification to confirm the generation of recombinant *B. subtilis*. MK, Takara DL 5000 DNA marker. Lanes 1 and 3, PCR amplification of *T. gondii* GRA12 and CotB-GRA12 fragments using wild-type *B. subtilis* genomic DNA as template. Lanes 2 and 4, PCR amplification of *T. gondii* GRA12 and CotB-GRA12 fragments using recombinant WB800n *B. subtilis* genomic DNA as template. **(E)** Western blotting analysis of the CotB-GRA12 fusion protein. MK, protein marker. Lanes1 and 2, loading buffer only. Lanes 3 and 4, coat proteins of wild-type *B. subtilis* spores. Lanes 5 and 6, coat proteins of recombinant *B. subtilis* spores (rBS-GRA12).

The CotB-GRA12 fusion gene was introduced into the WB800n genome via homologous recombination. The amyE gene in the plasmid replaced the amylase gene in the WB800n genome. **Figure 2A** is a flowchart representing the construction of recombinant *B. subtilis*. Recombinant and wild-type *B. subtilis* WB800n were inoculated onto solid LB medium containing 1% soluble starch. After overnight incubation, spraying with iodine solution resulted in medium around the recombinant WB800n colonies staining blue, whereas the medium around wild-type WB800n colonies did not (**Figures 2B, C**). To further demonstrate that inactivation of the amylase gene in recombinant *B. subtilis* WB800n was due to the insertion of an exogenous gene, DNA was extracted from wild-type and recombinant *B. subtilis* and subjected to PCR. CotB and GRA12 target fragments were successfully amplified in recombinant *B. subtilis*, whereas wild-type bacteria yielded no bands (**Figure 2D**). The coat proteins ofrBS-GRA12 and wild-type spores were also prepared and identified via western blotting method. CotB-GRA12 was successfully probed with mouse anti-GRA12 serum (lane 5, lane6), and there were no bands in the corresponding wild-type spores (lane 3, lane 4) and from the loading buffer only (lane 1, lane2) (**Figure 2E**). rBS-GRA12 was also identified via immunofluorescence assays. Compared to the negative control WB800n, a clear specific green fluorescent signal was detected on the surfaces of recombinant spores (**Supplementary Figure S2**), indicating that the fusion protein was successfully expressed and displayed on the spore surface.

### 3.3 Structure analysis of recombinant spores

**Figure 3:**
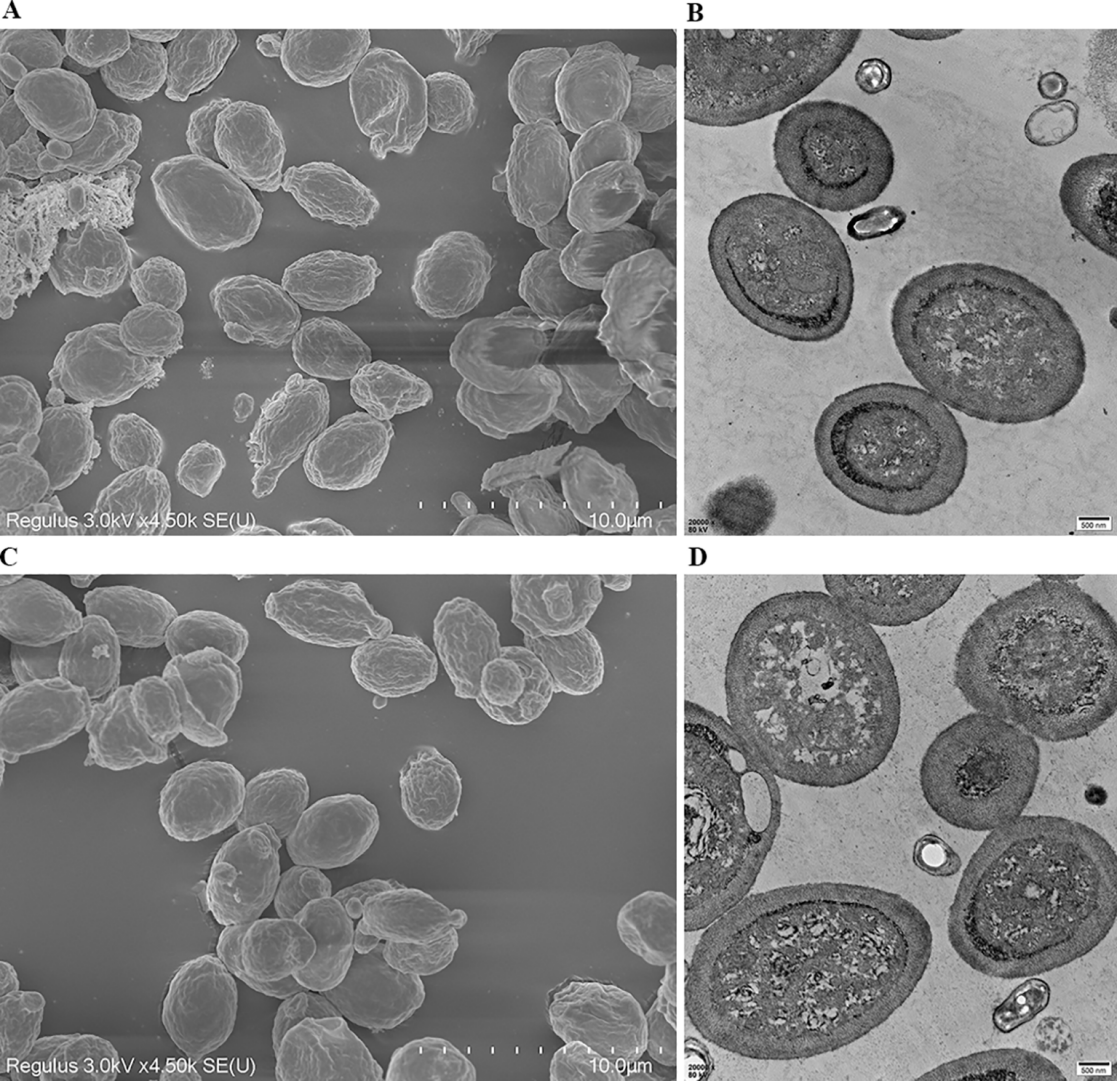
Ultrastructural analysis of wild-type and recombinant *B. subtilis* spores. The morphology and structure of recombinant *B. subtilis* spores (rBS-GRA12) were observed via **(A)** scanning electron microscopy and **(B)** transmission electron microscopy. The morphology and structure of the wild-type *B. subtilis* spores were also observed via **(C)** scanning electron microscopy and **(D)** transmission electron microscopy.

To determine whether expression of exogenous protein GRA12 affected the spore surface structure, purified spores were examined via SEM and TEM. There was no difference in the surface morphology of recombinant spores (**Figures 3A, B**) and wild-type spores (**Figures 3C, D**), indicating that expression of the exogenous protein GRA12 had no effect on the surface structure of the spores.

### 3.4 Analysis of splenic lymphocyte proliferation

**Figure 4:**
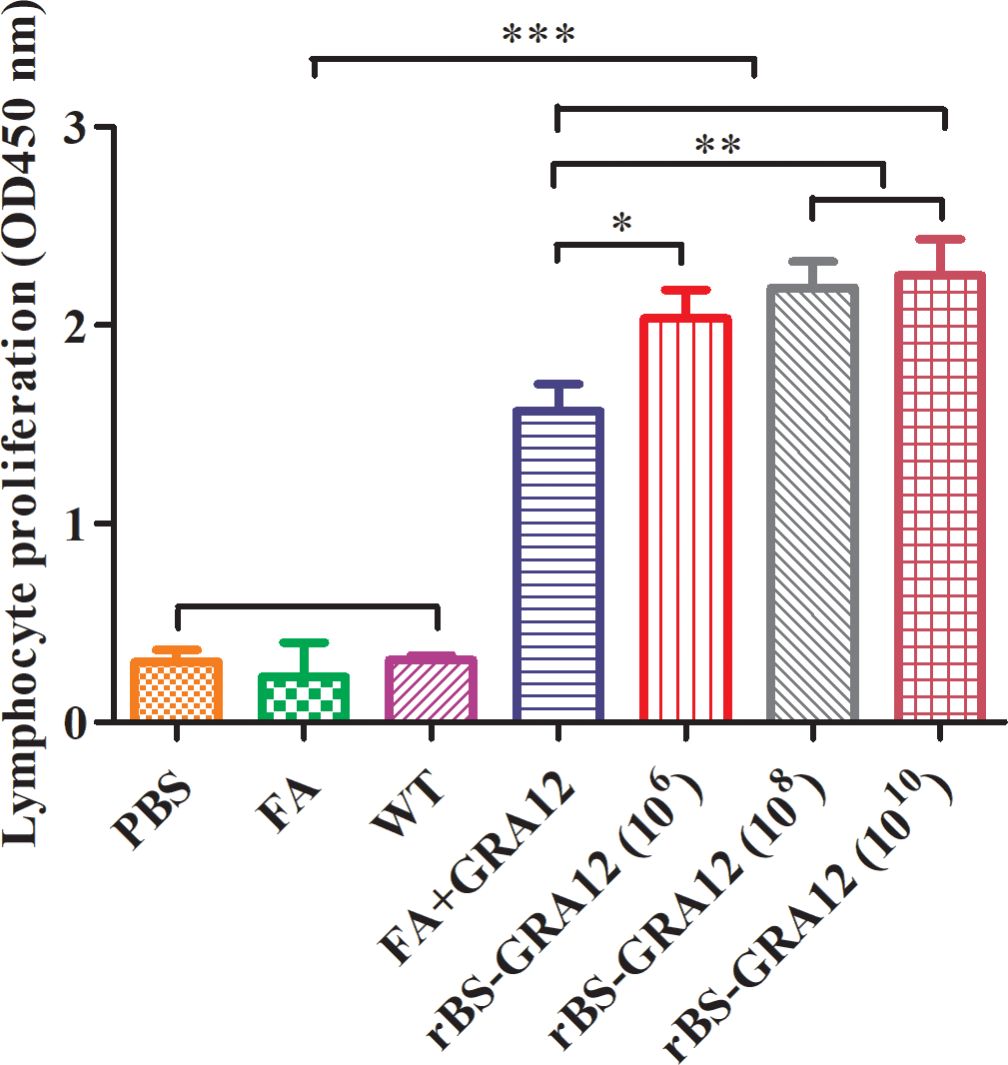
Splenic lymphocyte proliferation. Splenocyte proliferation was measured using the MTT method. The stimulation index (SI) was calculated as the ratio of the optical density (OD) of stimulated cells at 570 nm to the OD of unstimulated cells at 570 nm. One-way analysis and Student’s *t* test were used for statistical analyses. PBS, phosphate buffered saline; FA, Freund’s adjuvant; WT, wild-type *B. subtilis* spores; FA+GRA12, *T. gondii* GRA12 protein expressed in competent *E. coli* and emulsified with Freund’s adjuvant. rBS-GRA12, recombinant *B. subtilis* spores containing the *B. subtilis* CotB gene and the *T. gondii* GRA12 gene, where 10^6^, 10^8^, 10^10^ represent different inoculation doses calculated in colony-forming units. *P<0.05; **P<0.01; ***P<0.001.

Splenic lymphocytes proliferation in the recombinant spore groups and the FA+GRA12 group was significantly higher than that in the control groups (PBS, FA, and WT) (**Figure 4**). Proliferation in the recombinant spore groups was also significantly higher than that in the FA+GRA12 group (rBS-GRA12-10^6^ vs. FA+GRA12, *P*<0.05; rBS-GRA12-10^8^ and rBS-GRA12-10^10^ vs. FA+GRA12, *P*<0.01). There were no significant differences among the WT, PBS and FA groups (*P*>0.05).

### 3.5 Analysis of antibody levels

**Figure 5:**
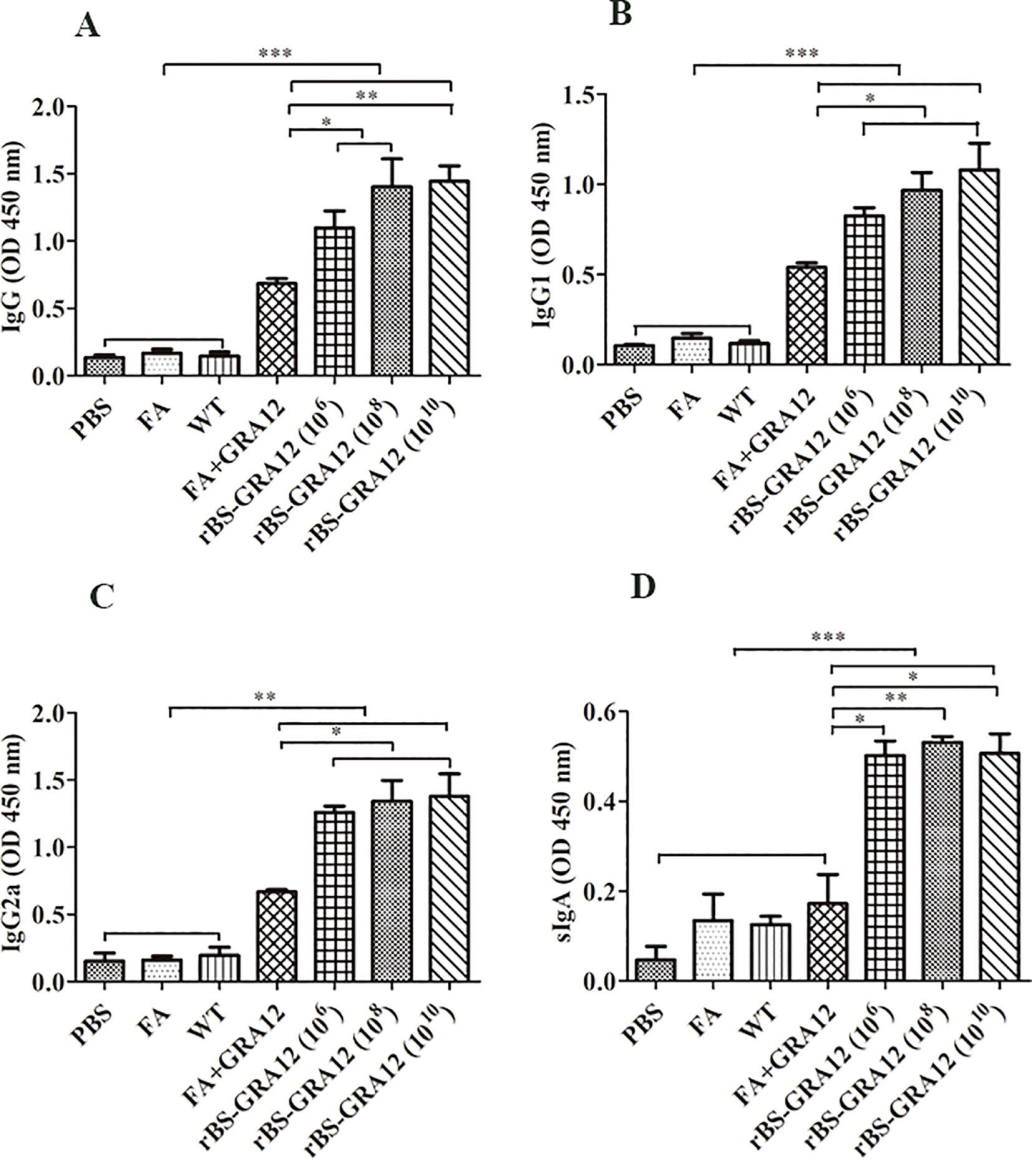
Evaluation of antibody expression levels. Total IgG, IgG1, IgG2a, and secretory IgA antibodies were detected via ELISA method. Samples were collected from three mice per group. **(A)** IgG titers. **(B)** IgG1 titers. **(C)** IgG2a titers. **(D)** Secretory IgA titers. One-way analysis of variance and Student’s *t* test were used for statistical analyses. *P<0.05; **P<0.01; ***P<0.001.

ELISAs indicated that GRA12 could significantly stimulate immune response. Higher IgG levels were detected in the rBS-GRA12 groups than in the FA+GRA12 group (rBS-GRA12-10^8^ vs. FA+GRA12, *P*<0.05; rBS-GRA12-10^10^ vs. FA+GRA12, *P*<0.01) and in the control groups (all *P*<0.001) (**Figure 5A**), indicating that immune responses generated by GRA12 were enhanced by the *B. subtilis* expression system. IgG1 and IgG2a subclasses were also assessed. Both were higher in the rBS-GRA12-10^8^ and rBS-GRA12-10^10^ groups than in the FA+GRA12 group (*P*<0.05), and IgG1 was significantly higher than in the control groups (*P*<0.001) (**Figures 5B, C**). Immunization with rBS-GRA12 induced significantly higher levels of IgG2a than those detected in the control groups (*P*<0.01). Similar results were evident in the FA+GRA12 group (*P*<0.05), but the IgG2a/IgG1 ratio was higher in the rBS-GRA12 group than in the FA+GRA12 group. Secretory IgA (sIgA) levels were significantly higher in the orally immunized groups than in the FA+GRA12 group (rBS-GRA12-10^6^ and rBS-GRA12-10^10^ vs. FA+GRA12, *P*<0.05; rBS-GRA12-10^8^ vs. FA+GRA12, *P*<0.01), and the control groups (*P*<0.001) (**Figure 5D**).

### 3.6 Cytokine assay

**Figure 6:**
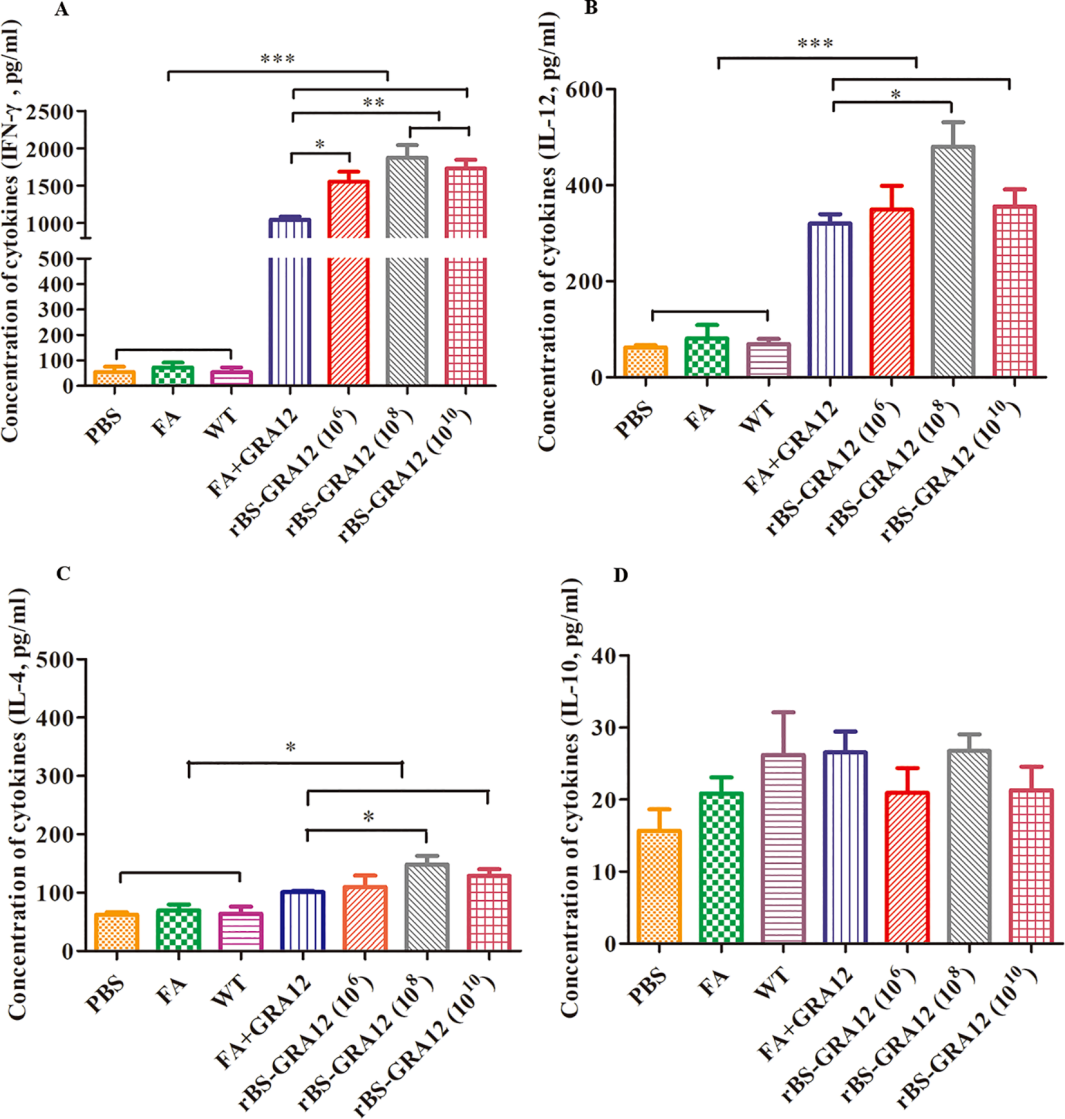
Analysis of cytokine expression levels. Splenic lymphocyte supernatant cytokine levels wwere evaluated via ELISAs. **(A)** IFN-γ. **(B)** IL-12. **(C)** IL-4. **(D)** IL-10. One-way analysis of variance and Student’s *t* test were used for statistical analyses. *P<0.05; **P<0.01; ***P<0.001.

IFN-γ and IL-12 levels were significantly elevated in the FA+GRA12 group and all three rBS-GRA12 groups compared to the control groups (*P*<0.001). Immunization with rBS-GRA12 (10^8^) and rBS-GRA12 (10^10^) increased the IFN-γ level significantly compared to FA+GRA12 immunization (*P*<0.01), and mice gavaged with rBS-GRA12 (10^6^) also exhibited higher levels of IFN-γ than the FA+GRA12 group (*P*<0.05). IL-12 levels in mice immunized with rBS-GRA12 (10^8^) were higher than those in the FA+GRA12 group (*P*<0.05) (**Figures 6A, B**). IL-4 levels in splenic lymphocyte supernatants were significantly increased in the vaccinated groups compared to the control groups (*P*<0.05), but there were no significant differences between the FA+GRA12 group and any of the rBS-GRA12 groups (**Figure 6C**). There were no significant differences in IL-10 expression levels among all of the groups (**Figure 6D**). Levels of IFN-γ, IL-12, IL-4, and IL-10 were also measured in sera from each group., and the results were essentially the same as those obtained in splenic lymphocyte culture supernatants (**Supplementary Table S2** in the **Supplementary Material**).

### 3.7 Quantification of brain and liver tissue parasite loads

Brain and liver parasite loads were measured in 3 mice from each group via absolute fluorescence quantitative PCR. Mice in all three rBS-GRA12 groups had significantly reduced parasite loads in the brain and liver compared to the control groups (*P*<0.01) (**Table 1**). Brain and liver parasite loads were significantly lower in the rBS-GRA12-10^8^ and rBS-GRA12-10^10^ immunized groups than in the FA+GRA12 group (*P*<0.05). Compared to WT immunized mice, brain parasite loads in the FA+GRA12 group were significantly reduced (*P*<0.05), and there were also significant differences compared to the PBS and FA groups (*P*<0.01).

**Table 1:** Brain and liver parasite loads in immunized BALB/c mice.

Immunized groups	Brain			Liver		
	CT value (mean± SD)	Parasite loads (mean)	Sig	CT value (mean± SD)	Parasite loads (mean)	Sig
PBS (1)	18.98 ± 1.40	1249624	(1vs4,5,6,7) b	20.23 ± 1.32	498055	(1vs4,5,6,7) b
FA (2)	18.84 ± 0.63	1118512	(2vs4,5,6,7) b	20.15 ± 1.21	517438	(2vs4,5,6,7) b
WT (3)	19.43 ± 1.53	958118	(3vs4) a (3vs,5,6,7) b	19.96 ± 1.27	569438	(3vs4,5,6,7) b
FA+GRA12 (4)	23.75 ± 0.65	26482	(4vs3,6,7) a (4vs1,2) b	24.74 ± 0.65	8385	(4vs6,7) a (4vs1,2,3) b
rBS-GRA12 (10^6^) (5)	24.95 ± 0.73	10821	(5vs1,2,3) b	26.24 ± 1.11	4593	(5vs1,2,3) b
rBS-GRA12 (10^8^) (6)	26.07 ± 1.02	8528	(6vs4) a (6vs1,2,3) b	28.76 ± 1.97	2661	(6vs4) a (6vs1,2,3) b
rBS-GRA12 (10^10^) (7)	26.08 ± 1.05	4987	(7vs4) a (7vs1,2,3) b	28.09 ± 1.88	1645	(7vs4) a (7vs1,2,3) b

^a^p<0.05.

^b^p<0.01.

### 3.8 Protection derived from rBS-GRA12

**Figure 7:**
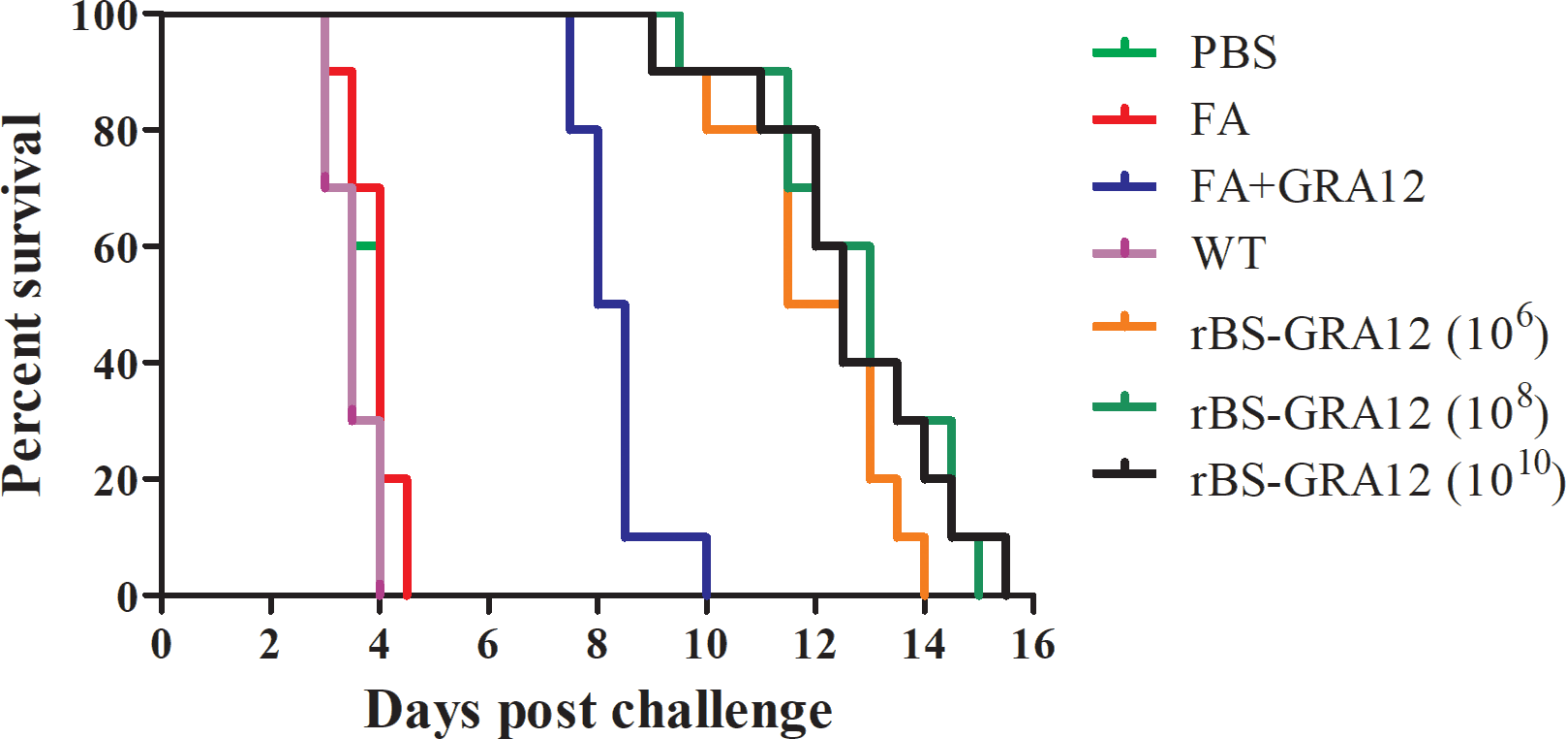
Survival curves of immunized mice after *T. gondii* infection. Survival times were monitored daily after challenge with *T. gondii* tachyzoites. The Kaplan–Meier approach was used to assess survival.

Some mice in the PBS, FA and WT groups became symptomatic and died on the third day after inoculation with *T. gondii*., and all have died by day 4.50. Some mice in the FA+GRA12 group died on day 7.50, and all had died by day 10.00, with a mean time to death of 8.75 d. Survival was significantly prolonged in the rBS-GRA12-10^6^, rBS-GRA12-10^8^ and rBS-GRA12-10^10^ groups, with mean survival times of 11.75 d, 12.25 d, and 12.00 d respectively (**Figure 7**). Mice in all three rBS-GRA12 groups exhibited significantly better survival times than the FA+GRA12 group (*P*<0.001). These results suggest that *B. subtilis* spores can partially enhance the immunoprotective effects of *T. gondii* GRA12.

## 4 Discussion

Recombinant live vaccines are genetically engineered to express pathogenic microorganism antigen via insertion into various types of genomes using viruses or bacteria as vectors. Similar to live attenuated vaccines, this type of vaccine can stimulate the host to produce a more effective immune response, produce antibodies in a short time, and provide long-lasting immunity (37, 38). The mucosal immune system plays a key role as the first line of defense against the external environment (39), and may contribute to resistance to most pathogenic microorganisms (40).

There is growing interest in the development of mucosal vaccines. Needle-free immunizations are safe, and also mimic natural infection and enhance immune system protection (41, 42). Novel approaches to enhance antigen-specific immune responses are new ideas for the development of effective mucosal vaccines. *B. subtilis* is widely used as an effective vaccine delivery system for the induction of mucosal immune responses, and has a unique effect on the immune system (43). *Escherichia coli* is now a more widely used expression system than *B. subtilis*, but there are many advantages to the *B. subtilis* expression system. *B. subtilis* spores can survive in the presence of high temperatures, lytic enzymes, toxic chemicals, and gastric acid (44).They are also non-pathogenic and non-invasive (45), and *B. subtilis* spore-based antigen display systems have demonstrated feasibility and efficacy (46). A clinical experiment was conducted with 16 participants who received oral *B. subtilis* spores expressing the S protein RBD of SARS-CoV-2 for three courses on three consecutive days. Participants who received the S protein spores showed an increase in neutralizing antibody levels against SARS-CoV-2, and no cases of local or systemic side effects were observed (47). As a powerful cellular and molecular tool, *B. subtilis* spores have a promising prospective in the development of vaccine delivery systems. However, there are still some critical issues that need to be addressed. Although *B. subtilis* spores are able to germinate in the intestine of host, it is not known whether the resporulation and growth are safe. Further study of proliferation mechanisms is essential for the development of safe and effective vaccines.

Humoral responses are well known to play an important role in immunity against *T. gondii*. Neutralizing antibodies are important indicator of vaccine efficacy, and serum IgG is the most abundant immunoglobulin in the blood and plays a critical role in systemic immune responses (48). IgG antibodies play an active role in both, the initiation as well as in the resolution phase of autoimmune inflammation. An IgG2a-dominant response indicates a Th1-type immune response, and IgG1 isotype titers in sera indicate a Th2-type immune response (49, 50). A good vaccine should induce protective cellular Th1 and humoral Th2 responses (15), and the capacity of *B. subtilis* spores to induce a balanced Th1 and Th2 response has been shown in previous studies (51). In the current study, repeated immunization of mice with rBS-GRA12 spores elicited increasing titers of IgG antibodies, with a Th1-Th2 combined immune response. One reason for this could be that as well as germinating in the mouse gut, *B. subtilis* spores appear to be able to grow and resporulate (52), and consequently continuously stimulate immune responses in the intestines (53). Zhang et al. (48) reported that a recombinant *B. subtilis* strain expressing the Porcine circovirus type 2 (PCV2) () capsid protein effectively triggered an immune response in newborn piglets by stimulating bone marrow-derived dendritic cell maturation and T cell proliferation. In another study, *B. subtilis* spores expressing the spike protein of Transmissible Gastroenteritis Virus could recruit dendritic cells and induce an immune response (54). IgA is the most abundant immunoglobulin in secretions. Secreted IgA (sIgA) is the product of local synthesis on the surfaces of the mucosal membranes, which are the major source of antigenic material for the body (55). SIgA has long been recognized as the first line of defense at the mucosal surface and is responsible for protecting against invading pathogens and toxins (56). In the present study, specific sIgA was induced by immunization with rBS-GRA12, indicating that the mucosal immune system was activated, and this is the first line of defense against *T. gondii* infection. Mucosal immunity is the primary defense against infection by pathogens, and sIgA can inhibit the adhesion and movement of pathogens (57).

In this study, serum IgG levels were higher than fecal IgA levels. Luiz et al. (58) reported that the use of an orally delivered *B. subtilis* vaccine expressing the structural subunit (CfaB) of the CFA/I fimbriae encoded by enterotoxigenic *Escherichia coli*, induced higher levels of serum IgG and fecal IgA antibodies in mice. Serum IgG levels were approximately 100 times higher than fecal IgA levels. Oral immunization with *B. subtilis* spores displaying the transmissible gastroenteritis virus (TGEV) spike protein could increase TGEV-specific secretory IgA in fecal and serum IgG antibodies in piglets, and the IgA response is very low compared to the IgG response (54). EI-Kamary et al. (59) reported that the induction of IgA responses after immunization with virus-like particle vaccine using Bespak intranasal delivery devices requires higher concentrations of antigen than those required to induce serum IgG in mice, and it is not yet known whether this possibility exists for oral vaccination. In the present study, a direct comparison between serum IgG and fecal IgA has not yet been carried out, but this should be included in future studies.

Cellular immunity is an important defense mechanism against intracellular pathogens. Continuous production of IFN-γ is required to control both acute and chronic *T. gondii* infection (60). IL-12 is essential for activating natural killer cells or inducing type 1 T cell proliferation, and subsequently induces the production of IFN-γ (61). Th2 immune responses are characterized by IL-4 (50), which can promote cytotoxic T cell activity and enhance phagocytic cell function (62). Zhang et al. (48) reported that IFN-γ was significantly increased in piglets immunized with *B. subtilis-*Cap, and that vaccine could be administered orally, and it induced more robust humoral and cellular immunity than inactivated PCV2. The increased production of IFN-γ, IL-12, and IL-4 by splenic lymphocyte in the current study is likely to indicate an integrated Th1/Th2 immune response. Some studies suggest that recombinant *B. subtilis* spores have adjuvant-like properties. They can enhance the immune response by promoting efficient and direct antigen presentation via MHC I/II, leading to balanced Th1 and Th2 responses (51, 63, 64).

The level of lymphocyte proliferation has a direct effect on the level of the body’s immunity, particularly cellular immunity (65). In the present study, splenic lymphocyte proliferation was markedly increased in mice after oral immunization with recombinant spores expressing GRA12 protein. Hu et al. (66) reported that the splenic lymphocyte proliferation simulation index (SI) in mice immunized with recombinant *B. subtilis* expressing the B subunit of cholera toxin and an epitope box from foot-and-mouth disease virus was higher than the SI induced by a commercial vaccine. Yang et al. (67) reported higher levels of lymphocyte proliferation in rBS*^CotB-HcG^* -immunized mice than in control mice and mice vaccinated with recombinant *Haemonchus contortus* GAPDH protein. The ratio of CD4^+^ to CD8^+^ cells is an important factor and plays a central role in the induction of effective immune responses (68). In future immunological experiments, we will measure the number of CD4^+^ and CD8^+^ lymphocytes for a more comprehensive assessment of vaccine immunity effect. *B. subtilis* spores induced stronger recall responses than a commercial oil adjuvant-based vaccine, and they may have advantages as adjuvants for generating antigen-specific memory responses (69). Oral immunization has the ability to interact with intestinal epithelial cells and stimulate the proliferation of gut-associated lymphocytes. Mauriello et al. reported that recombinant *B. subtilis* spores displaying the C fragment of the tetanus toxin induced spleen and mesenteric lymph nodes cell proliferation (70). The intestinal immune system is the most complex immune system. The immune responses in the gut are initiated in organized lymphoid organs, including Peper’s patches (PPs) and mesenteric lymph nodes (MLNs). The MLNs stand out as the most important lymph nodes in the human body, crucial for regulating the peripheral and mucosal recirculation pathways (71). Among the various local lymphoid tissues, the MLNs appear to be the primary site where oral tolerance is predominantly induced. Presentation of fed antigens occurs more frequently in the MLNs compared to PPs (72). The dendritic cells (DCs) migrate from the PPs or epithelium to the MLNs in association with other antigen-presenting cells, and then the native T-cells are activated (73). Our results provide preliminary evidence that *B. subtilis* spores enhance the immunoprotective effects of GRA12 by inducing systemic immunity. The molecular mechanisms by which *B. subtilis* enhances the immune response will be the focus of future research and we will explore local immune response mechanisms.

In the present study, oral administration of rBS-GRA12-10^8^ and rBS-GRA12-10^10^ resulted in significantly higher levels of splenic lymphocytes proliferation compared to the rBS-GRA12-10^6^ group. Our results were consistent with those reported by Tang et al. (74), who reported that oral immunization of recombinant *B. subtilis* spores expressing cysteine protease of *Clonorchis sinensis* (*C. sinensis*, *B.s-CsCP*) in mice could increase system immune levels in a dose-dependent manner. Although humoral responses were increased in rBS-GRA12-10^8^ and rBS-GRA12-10^10^ immunized mice compared to rBS-GRA12-10^6^ immunized mice, they were not statistically significant. The levels of sIgA expression were essentially the same in these three groups. There was no significant difference in antibody levels between the rBS-GRA12-10^8^ and rBS-GRA12-10^10^ groups. Although the rBS-GRA12-10^8^ and rBS-GRA12-10^10^ groups induced a statistically significant higher immune response than the rBS-GRA12-10^6^ group, there was no statistically significant difference between the rBS-GRA12-10^8^ and rBS-GRA12-10^10^ groups. The molecular mechanisms by which recombinant spores activate the immune response at different doses are not known. Therefore, we chose to use these three doses in subsequent immunoprotection experiments. The dosage of rBS-GRA12-10^8^ will be selected for use in future studies and higher spore concentrations are unnecessary and increase production costs.

Direct approaches to evaluating the protective efficacy of a vaccine candidate against acute and chronic toxoplasmosis include comparing survival times and parasite loads in vaccinated mice (75). The majority of antigens administered orally are poorly immunogenic and are degraded by gastric acid and hydrolysis of proteins in the gastrointestinal tract. *B. subtilis* spores that enable it to resist complex environments and stimulate effective immunoprotective responses (24). In the present study, FA+GRA12 was injected intraperitoneally to avoid its degradation, whereas *B. subtilis* spores were immunized by oral administration. Mice in all three rBS-GRA12 groups exhibited longer survival times than mice in the FA+GRA12 group, indicating that the recombinant *B. subtilis* spores induced immune responses and significantly enhanced the immunoprotective capacity of GRA12. Although mice immunized with rBS-GRA12 exhibited improved resistance to high virulent *T. gondii* strain infection, the vaccine did not provide complete protection. However, it is still superior to other single-gene DNA vaccines in terms of survival time. Immunization with the vaccine encoding *T. gondii* SAG4 could trigger strong immune response, and the survival time in BALB/c mice was 9.3 ± 1.64 days (76). GRA16 DNA vaccine dramatically increased IL-2, IFN-γ and IL-10 cytokine levels, while a marginal increase in IL-4 was observed. Although their improvements did not help to prolong the survival of the immunized mice, a significant reduction in the number of cysts in the brain was observed (77). Immunization with single *T. gondii* stage-specific antigen has been shown to provide limited protection, likely due to the complexity of *T. gondii* life cycle. The use of a multimeric vaccination approach, incorporating B and T cell epitopes of different parasite life cycle stages, may constitute a more potent, durable means of preventing toxoplasmosis (78). In our future research, analysis of other protective toxoplasmosis antigens will be investigated in combination with that used in the present study. Mice immunized with rBS-GRA12 exhibited significant reduction in parasite loads in the brain and liver compared to mice immunized with GRA12 alone. To evaluate parasite loads, brain and liver tissues were collected from three mice in each group that exhibited clinical symptoms but did not die. Although the numbers of parasites in the tissues of mice immunized with rBS-GRA12 were drastically lower, the vaccine did not provide complete protection against challenge with the RH strain. The main reason for this is that the immune protection provided by a single antigen is more limited. These results indicate that the rBS-GRA12-based vaccine was successful in activating components of the immune system, and represents viable strategy for the development of *T. gondii* vaccine. It has previously been reported that a combination of *B. subtilis* and coccidiosis vaccines could improve protection against *Eimeria* spp. infection (79). In another study all mice immunized with RSM2e3 spores survived after challenge with an H1N1 influenza virus (53), indicating the capacity of the recombinant spores to induce a protective immune response.

## 5 Conclusion

In the current study, recombinant *B. subtilis* spores based on the *T. gondii* GRA12 protein were successfully constructed, and the immunogenicity and protective efficacy of rBS-GRA12 were evaluated for the first time. Immunization of mice with rBS-GRA12 resulted in significantly prolonged survival time and a marked reduction in the numbers of parasites in brain and liver tissues after *T. gondii* infection. The results of the present study provide new ideas and a theoretical basis for the development of new *T. gondii* vaccines. The research performed in the current study did not assess the immune mechanisms by which *B. subtilis* spores enhanced the immunological effects of the GRA12 protein, and further studies are needed to better understand the immune mechanisms involved.

## Data Availability

The original contributions presented in the study are included in the article/**Supplementary Material**. Further inquiries can be directed to the corresponding author/s.

## Supplementary material

Supplementary Figure 1 A diagram of the different immunizations.

Supplementary Figure 2 Immunofluorescence microscopy of the recombinant and wild-type *B. subtilis* spores. The spores were incubated with the mouse anti-GRA12 antibody, followed by an incubation with the anti-mouse IgG-Alexa Fluor 488-conjugate. **(A, B)** Immunofluorescence and bright field of recombinant *B. subtilis* spores. **(C, D)** Immunofluorescence and bright field of wild-type *B. subtilis* spores. The scale bar represents 1 μm.
