# Supplementary figures and images for: Bacillus subtilis spores displaying Toxoplasma gondii GRA12 induce immunity against acute toxoplasmosis
Source: Front Immunol. 2025 Feb 26;16:1457560. doi: 10.3389/fimmu.2025.1457560 (PMC11897052; doi:10.3389/fimmu.2025.1457560)

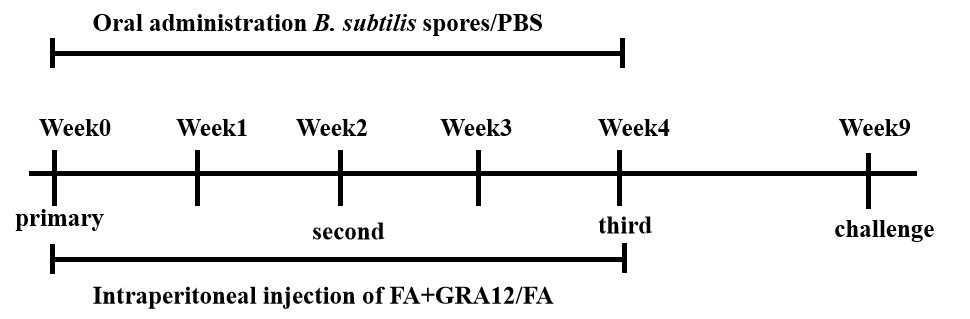

Supplement: Supplementary Figure 1 — A diagram of the different immunizations. [file Image1.tif]

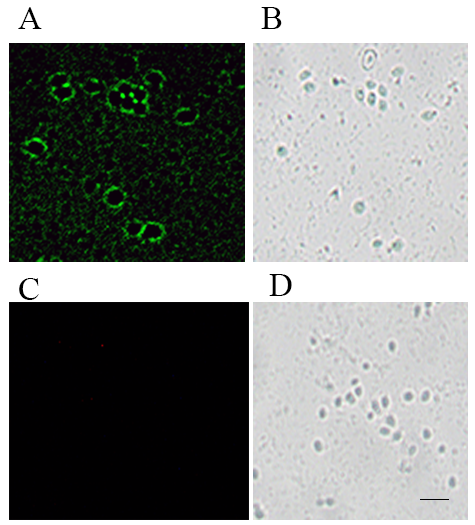

Supplement: Supplementary Figure 2 — Immunofluorescence microscopy of the recombinant and wild-type B. subtilis spores. The spores were incubated with the mouse anti-GRA12 antibody, followed by an incubation with the anti-mouse IgG-Alexa Fluor 488-conjugate. (A, B) Immunofluorescence and bright field of recombinant B. subtilis spores. (C, D) Immunofluorescence and bright field of wild-type B. subtilis spores. The scale bar represents 1 μm. [file Image2.tif]
