# Supplementary material for: Bacillus subtilis spores displaying Toxoplasma gondii GRA12 induce immunity against acute toxoplasmosis
Source: Front Immunol. 2025 Feb 26;16:1457560. doi: 10.3389/fimmu.2025.1457560 (PMC11897052; doi:10.3389/fimmu.2025.1457560)
Supplement: Supplementary file 3 [file Table1.doc]

**Table S1 The immunization process of BALB/c mice**

| Immunization  groups | Immunization time ^a^ | | | Immunization  method |
| --- | --- | --- | --- | --- |
|  | First time | Second time | Third time |  |
| PBS | 100 μl | 100 μl | 100 μl | oral |
| FA | 100 μl  (Freund's complete adjuvant) | 100 μl  (Freund's incomplete adjuvant) | 100 μl  (Freund's incomplete adjuvant) | intraperitoneal |
| FA+GRA12 | 200 μg | 100 μg | 100 μg | intraperitoneal |
| WT (10^10^) | 10^10^ CFU | 10^10^ CFU | 10^10^ CFU | oral |
| rBS-GRA12 (10^6^) | 10^6^ CFU | 10^6^CFU | 10^6^ CFU | oral |
| rBS-GRA12 (10^8^) | 10^8^CFU | 10^8^CFU | 10^8^CFU | oral |
| rBS-GRA12 (10^10^) | 10^10^ CFU | 10^10^ CFU | 10^10^ CFU | oral |

^a^ All mice were immunized (oral or intraperitoneal) three times at one week interval.
