# Supplementary material for: Bacillus subtilis spores displaying Toxoplasma gondii GRA12 induce immunity against acute toxoplasmosis
Source: Front Immunol. 2025 Feb 26;16:1457560. doi: 10.3389/fimmu.2025.1457560 (PMC11897052; doi:10.3389/fimmu.2025.1457560)
Supplement: Supplementary file 4 [file Table2.doc]

**Table S2 Cytokine production in serum from** BALB/c mice

| Immunization  groups | Cytokine production (pg/mL) | | | |
| --- | --- | --- | --- | --- |
|  | IFN-γ | IL-12 | IL-4 | IL-10 |
| PBS | 110.95±8.46 | 87.35±4.60 | 52.08±4.32 | 89.16±4.22 |
| FA | 127.18±14.19 | 102.46±14.18 | 66.74±15.26 | 95.14±10.46 |
| FA+GRA12 | 946.85±8.46_a_ | 340.94±15.28_d_ | 114.68±6.62_f_ | 102.08±6.64 |
| WT (10^10^) | 122.73±10.66 | 118.92±7.41 | 78.34±8.26 | 98.49±4.02 |
| rBS-GRA12 (10^6^) | 1246.73±65.37_a, b_ | 358.96±22.43_d_ | 131.38±10.06_f_ | 96.91±8.02 |
| rBS-GRA12 (10^8^) | 1662.02±102.18_a, c_ | 462.33±16.09_d, e_ | 166.85±17.73_f, g_ | 106.32±4.46 |
| rBS-GRA12 (10^10^) | 1509.32±69.17_a, c_ | 386.73±47.07_d_ | 146.91±8.57_f_ | 98.07±3.27 |

a, compared with PBS, FA or WT (10^10^), *p* < 0.001.

b, e, g, compared with FA+GRA12, *p* < 0.05.

c, compared with FA+GRA12, *p* < 0.01.

d, compared with PBS, FA or WT (10^10^), *p* < 0.01.

f, compared with PBS, FA or WT (10^10^), *p* < 0.05.
